# Context-dependent reprogramming of *ALS* adhesin expression in *Candida albicans*: a multi-inducer analysis linking morphogenesis to virulence plasticity

**DOI:** 10.3389/fmicb.2026.1848556

**Published:** 2026-06-29

**Authors:** Radfan Ahmed, Arvind Kayande, Rajendra Patil, Amruta Shelar, Gajanan B. Zore

**Affiliations:** 1 School of Life Sciences, Swami Ramanand Teerth Marathwada University, Nanded, Maharashtra, India; 2 Faculty of Medicine and Health Sciences, Albaydha University, Albaydha, Yemen; 3 Department of Biotechnology, School of Life Sciences, Central University of Rajasthan, Kishangarh, India; 4 Department of Biotechnology, Savitribai Phule Pune University, Pune, Maharashtra, India; 5 Department of Technology, Savitribai Phule Pune University, Pune, Maharashtra, India

**Keywords:** adhesins, ALS gene family, anti-adhesion therapy, biofilm formation, *Candida albicans*, morphogenesis, temporal gene regulation, virulence factors

## Abstract

*Candida albicans* employs the *ALS* (Agglutinin-Like Sequence) gene family to encode cell-surface adhesins that are central to host colonization, tissue invasion, and biofilm formation. Although individual *ALS* genes have been studied under specific conditions, no systematic, multi-inducer temporal analysis of the entire family has been reported to date. In this study, we present the first comprehensive temporal expression profiling of seven *ALS* genes (*ALS1*-*ALS5*, *ALS7*, and *ALS9*) under six host-relevant inducers, temperature (37 °C), neutral pH, serum, glucose, *N*-acetylglucosamine (NAG), and proline, across four time points (45 min, 90 min, 3 h, 6 h) in both yeast (30 °C) and hyphal (37 °C) growth phases. Morphological analysis confirmed that 37 °C and serum were the most potent hyphal inducers (94.13 ± 0.94% hyphae with serum at 37 °C). Quantitative PCR revealed a temporally stratified regulatory program: early-phase adhesins (*ALS1*, *ALS2*) showed transient induction during the first 90 min, whereas invasion-associated genes exhibited sustained late-phase activation. Notably, *ALS3* displayed exceptional upregulation under glucose at 37 °C (25.17 ± 1.76-fold at 6 h; *p* < 0.001) and responded robustly to all inducers except serum. *ALS7* reached 9.48-fold induction specifically under thermal stress, while *ALS5* was preferentially expressed at 30 °C (6.70-fold under neutral pH), indicating niche-specific functional specialization. *In silico* promoter analysis linked these expression patterns to binding motifs for key transcription factors (Efg1, Cph1, Rim101, and Nrg1). Phylogenetic analysis revealed functional conservation of *ALS2*/*ALS4* versus evolutionary divergence in *ALS6*/*ALS7*/*ALS9*. STRING-based protein interaction networks confirmed the central involvement of ALS proteins in adhesion and biofilm regulatory circuits. These findings establish a molecular framework for niche-specific virulence, identifying *ALS3* as a prime therapeutic target for anti-adhesion strategies in invasive candidiasis. We hypothesize that *C. albicans* employs a temporally stratified and inducer-specific transcriptional program of the ALS family to adapt its surface architecture to diverse host microenvironments.

## 1 Introduction

*Candida albicans* is the most prevalent human fungal pathogen, responsible for a spectrum of infections ranging from superficial mucocutaneous candidiasis to life-threatening systemic disease in immunocompromised individuals (Mayer et al., 2013; Katsipoulaki et al., 2024). The pathogenic potential of this organism is intimately tied to a suite of virulence attributes, among which adhesion to host surfaces represents the critical first step in establishing infection (Calderone and Fonzi, 2001; Hoyer et al., 2016). Among the adhesin repertoire of *C. albicans*, the Agglutinin-Like Sequence (*ALS*) gene family occupies a central position, encoding eight large cell-surface glycoproteins (*ALS1*p-*ALS7*p and *ALS9*p) that mediate attachment to a diverse array of host ligands including fibronectin, laminin, collagen, E-cadherin, and *N*-cadherin (Hoyer et al., 2008; Zhu and Filler, 2010; Hoyer et al., 2016).

The eight *ALS* genes (*ALS1*-*ALS7* and *ALS9*) are distributed across distinct chromosomal loci, a genomic arrangement that likely facilitates their independent regulatory control during environmental adaptation, and share a conserved structural organization: an *N*-terminal adhesive domain, a central tandem repeat region of variable length, and a C-terminal stalk domain that anchors the protein to the cell wall via glycosylphosphatidylinositol (GPI) linkages to *β*-1,6-glucan (Hoyer et al., 2008; Hoyer et al., 2016; Hoyer and Als, 2001). Despite this shared architecture, individual *ALS* genes display distinct expression profiles depending on morphological state, growth conditions, and the host niche colonized (Green et al., 2004; Cheng et al., 2005; Zhao et al., 2005; Argimón et al., 2007).

The reversible transition between yeast and hyphal morphologies is a defining virulence trait of *C. albicans*, with hyphae playing essential roles in tissue invasion, immune evasion, and biofilm architecture (Sudbery, 2011; Basso et al., 2018; Wakade et al., 2024). This morphological switch is triggered by host-relevant environmental signals including elevated temperature (37 °C), neutral-to-alkaline pH, serum, and specific carbon and nitrogen sources and is governed by interconnected signalling cascades such as the Ras1-cAMP-PKA, MAPK, and Rim101 pathways, converging on key transcription factors including Efg1, Cph1, Nrg1, Tup1, and Bcr1 (Kadosh, 2019; Glazier, 2022; Wakade et al., 2023). Among the *ALS* family, *ALS3* has attracted particular attention as a hypha-specific invasin that binds E-cadherin and *N*-cadherin to promote endocytosis by host epithelial and endothelial cells (Phan et al., 2007; Liu and Filler, 2011).

Despite two decades of research on individual *ALS* genes, a fundamental gap persists in our understanding of how the entire *ALS* family is temporally regulated across multiple host-relevant conditions within a single experimental framework. Prior studies have examined *ALS* expression in reconstituted human epithelium (Green et al., 2004), clinical vaginal specimens (Cheng et al., 2005; Monroy-Pérez et al., 2012), or single-inducer settings, but none have systematically mapped the temporal dynamics of *ALS* gene expression across multiple environmental cues. Such information is critical because *C. albicans* encounters a mosaic of microenvironments during infection, and the adhesin profile it presents to the host must be dynamically tailored to each niche.

In this study, we address this knowledge gap by presenting the first comprehensive, multi-inducer temporal analysis of *ALS* gene family expression in *C. albicans*. We profiled the expression of seven *ALS* genes (*ALS1-ALS5*, *ALS7*, *and ALS9*) under six host-relevant inducers (temperature, neutral pH, serum, glucose, NAG, and proline) at four time points spanning the yeast-to-hypha transition. *ALS6* was intentionally excluded from this study: prior literature consistently reports that *ALS6* is expressed at low, non-differential levels under a broad range of conditions and contributes minimally to adhesion relative to other family members (Hoyer & Hecht, 2001). This experimental design was complemented by *in silico* analyses of promoter architecture, phylogenetic relationships, protein interaction networks, and domain structure. Our findings reveal a biphasic regulatory program with early adhesins mediating initial attachment and late-phase effectors driving invasive growth and identify condition-specific vulnerabilities that may be exploited for targeted anti-adhesion therapies.

## 2 Materials and methods

### 2.1 Chemicals, reagents, and growth media

All fine chemicals and reagents were procured from Sigma-Aldrich Chemicals (India) Pvt. Ltd., Bangalore. Growth media and components were obtained from HiMedia Laboratories Pvt. Ltd., Mumbai, India.

### 2.2 Microbial culture and inoculum preparation

*Candida albicans* (ATCC 10231) was obtained from the Microbial Type Culture Collection (MTCC), Institute of Microbial Technology (IMTech), Chandigarh, India, and maintained on YEPD agar slants (1% yeast extract, 2% peptone, 2% dextrose, 2.5% agar, pH 6.5) at 28 °C with storage at 4 °C. For inoculum preparation, cells were cultured in YEPD broth at 30 °C for 24 h, harvested by centrifugation (1,000 rpm, 2 min), washed thrice with sterile distilled water, and starved for 1 h at 30 °C. Cell density was adjusted to 1 × 10^7^ cells/ml using a haemocytometer (Zore et al., 2011). The starvation step was performed to synchronize the metabolic state of the cells and minimize any carry-over effects from the rich YEPD medium, thereby ensuring that observed gene expression changes are primarily driven by the experimental induction conditions.

### 2.3 Induction of filamentous growth

Filamentous growth was induced using six host-relevant environmental conditions. Starved cells (1 × 10^7^ cells/ml) were inoculated into pre-warmed (37 °C) YEPD-based induction media under the following conditions: (i) elevated temperature (37 °C) in YEPD at pH 6.5; (ii) neutral pH (pH 7.0) in YEPD at 37 °C; (iii) 10% foetal bovine serum in YEPD at pH 6.5; (iv) 5 mM glucose in YEPD at pH 6.5; (v) 2.5 mM *N*-acetylglucosamine (NAG) in YEPD at pH 6.5; and (vi) 2.5 mM proline in YEPD at pH 6.5. All treatments were incubated at 37 °C. Control cultures were maintained at 30 °C. Each condition was tested in biological triplicate. Cultures were sampled at 45 min, 90 min, 3 h, and 6 h for RNA isolation. The percentage of hyphal induction was assessed microscopically at 90 min for serum and at 3 h for all other conditions. Hyphae and pseudohyphae were quantified microscopically by observing at least 200 cells per field from three independent fields for each sample. Hyphae were defined as germ tubes with parallel-sided walls and a length greater than twice the diameter of the mother yeast cell. Pseudohyphae were characterized by constricted septa and elongated yeast-like cells forming chains.

### 2.4 RNA extraction and real-time quantitative PCR

Total RNA was isolated from 1 × 10^8^ cells (or equivalent hyphal biomass) under each condition. Cells were lysed with 300 units of lyticase in the presence of 1% *β*-mercaptoethanol in SG buffer. RNA was extracted using the RNA Sure Mini Kit (Nucleo-pore, Genetix) following the manufacturer’s protocol. One microgram of total RNA was reverse-transcribed using the SuperScript First-Strand Synthesis System (Invitrogen). Quantitative PCR was performed on a CFX96 Touch™ Real-Time PCR Detection System (Bio-Rad) using KAPA SYBR® FAST qPCR Master Mix (2X) Universal with gene-specific primers for *ALS1-ALS5*, *ALS7*, *ALS9*, and the reference gene *GAPDH* (Table 1; primers from (Green et al., 2004). Relative gene expression was calculated using the 2^-ΔΔCt^ method (Livak and Schmittgen, 2001; Schmittgen and Livak, 2008), normalised first to *GAPDH* and then to untreated control samples at 30 °C. All reactions were performed in triplicate. GAPDH Ct values varied by less than 0.5 cycles across all conditions tested, confirming its suitability as a reference gene and satisfying MIQE guideline requirements (Bustin et al., 2009). Data are presented as mean ± standard deviation (SD; *n* = 3). Statistical significance was assessed by Welch’s *t*-test (two-tailed, unequal variances) comparing 30 °C versus 37 °C expression at each time point independently, which is the appropriate pairwise test for this experimental design with *n* = 3 per group. A fold-change threshold of ≥1.5 (induction) or ≤0.5 (repression) was applied as the primary criterion for biologically meaningful differential expression, consistent with established qPCR reporting standards (Bustin et al., 2009). Significance levels: *** *p* < 0.001; ** *p* < 0.01; * *p* < 0.05.

**Table 1:** Oligonucleotide primers used for RT-qPCR analysis of the *ALS* gene family (adapted from Green et al., 2004).

Gene	Gene ID	Accession No.	Forward primer (5′ → 3′)	Reverse primer (5′ → 3′)	Size (bp)
*ALS1*	3,640,280	L25902	GACTAGTGAACCAACAAATACCAGA	CCAGAAGAAACAGCAGGTGA	318
*ALS2*	3,646,295	AF024580	CCAAGTATTAACAAAGTTTCAATCACTTAT	TCTCAATCTTAAATTGAACGGCTTAC	366
*ALS3*	3,647,969	U87956	CCACTTCACAATCCCCATC	CAGTAGTAGTAACAGTAGTAGTTTCATC	120
*ALS4*	3,647,972	AF024584	CCCAGTCTTTCACAAGCAGTAAAT	GTAAATGAGTCATCAACAGAAGCC	356
*ALS5*	3,640,334	AY227440	TGACTACTTCCAGATTTATGCCGAG	ATTGATACTGGTTATTATCTGAGGGAGAAA	318
*ALS7*	3,642,234	AF201684	GAAGAGAACTAGCGTTTGGTCTAGTTGT	TGGCATACTCCAATCATTTATTTCA	206
*ALS9*	3,640,316	AF229990	CCATATTCAGAAACAAAGGGTTC	AACTGAAACTGCTGGATTTGG	198

### 2.5 *In silico* analyses

Promoter sequences (1,000 bp upstream of the start codon) for all *ALS* genes in *C. albicans* SC5314 were retrieved from the *Candida* Genome Database (CGD; https://www.candidagenome.org/). Transcription factor binding sites were predicted using the MEME Suite (v5.5.1) and YEASTRACT+ databases, focusing on morphogenesis-related regulators (Efg1, Cph1, Nrg1, Tup1, Brg1, Rim101). Phylogenetic relationships among *ALS* family members in *C. albicans* SC5314 and *C. dubliniensis* CD36 were evaluated using neighbour-joining trees constructed in MEGA X with bootstrap analysis (1,000 replicates). Gene Ontology enrichment was performed using g: Profiler, and protein–protein interaction networks were analysed via the STRING database (v12.0) with a medium confidence threshold (≥0.400). ALS protein domain architecture was predicted using Pfam (v35.0), SMART, and MobiDBLite.

## 3 Results

### 3.1 Environmental inducers elicit distinct Morphogenic responses

Quantitative morphological analysis established 37 °C as the principal driver of hyphal development in *C. albicans* ATCC 10231. Under basal conditions at 30 °C, the culture was dominated by budded yeast cells (60.76 ± 1.22%), with no detectable hyphae. Elevation to 37 °C alone induced 61.12 ± 1.57% hyphal formation (*p* < 0.001). Among the six inducers tested, serum at 37 °C provoked near-complete hyphal conversion (94.13 ± 0.94%), followed by glucose (88.93 ± 1.34%), proline (88.02 ± 1.21%), NAG (87.92 ± 1.01%), and neutral pH (79.43 ± 0.89%). At 30 °C, serum was the only inducer that generated appreciable morphological change, inducing a pseudohyphal-dominant phenotype (52.00 ± 1.19%) rather than true hyphae (Table 2). These observations confirm a tiered regulatory hierarchy in which physiological temperature establishes competence for filamentation, while specific environmental signals modulate its extent and character.

**Table 2:** Morphogenic response of *C. albicans* to environmental inducers.

Inducer	Temp.	Hyphae (%)	Pseudohyphae (%)	Budded (%)	Unbudded (%)
Temperature	30 °C	0.00 ± 0.0	1.30 ± 0.3	60.76 ± 1.22	38.53 ± 1.32
	37 °C	61.12 ± 1.57***	3.34 ± 0.57	22.09 ± 1.18***	13.45 ± 1.54***
pH7.0	30 °C	6.06 ± 0.82	2.16 ± 0.78	56.33 ± 1.32	34.13 ± 1.41
	37 °C	79.43 ± 0.89***	2.57 ± 0.55	11.97 ± 0.79***	6.04 ± 0.91***
Serum (10%)	30 °C	31.56 ± 1.01	52.00 ± 1.19	14.44 ± 1.19	1.99 ± 0.52
	37 °C	94.13 ± 0.94***	0 ± 0.00	4.16 ± 0.65***	1.72 ± 0.33
Glucose (5 mM)	30 °C	8.37 ± 0.96	6.26 ± 0.95	63.13 ± 1.06	22.88 ± 0.89
	37 °C	88.93 ± 1.34***	1.35 ± 0.33	5.30 ± 0.89***	4.41 ± 0.71***
NAG (2.5 mM)	30 °C	12.91 ± 0.88	7.99 ± 0.92	57.49 ± 1.35	21.51 ± 1.35
	37 °C	87.92 ± 1.01***	1.78 ± 0.32	8.50 ± 1.45***	2.23 ± 0.62***
Proline (2.5 mM)	30 °C	6.07 ± 1.05	6.54 ± 1.21	65.94 ± 1.47	21.88 ± 0.94
	37 °C	88.02 ± 1.21***	0.46 ± 0.00	10.58 ± 1.40***	0.95 ± 0.30***

Values represent mean ± SD of three independent experiments. ***p < 0.001 versus 30 °C control.

### 3.2 Temperature-dependent regulation of *ALS* gene expression

**Figure 1:**
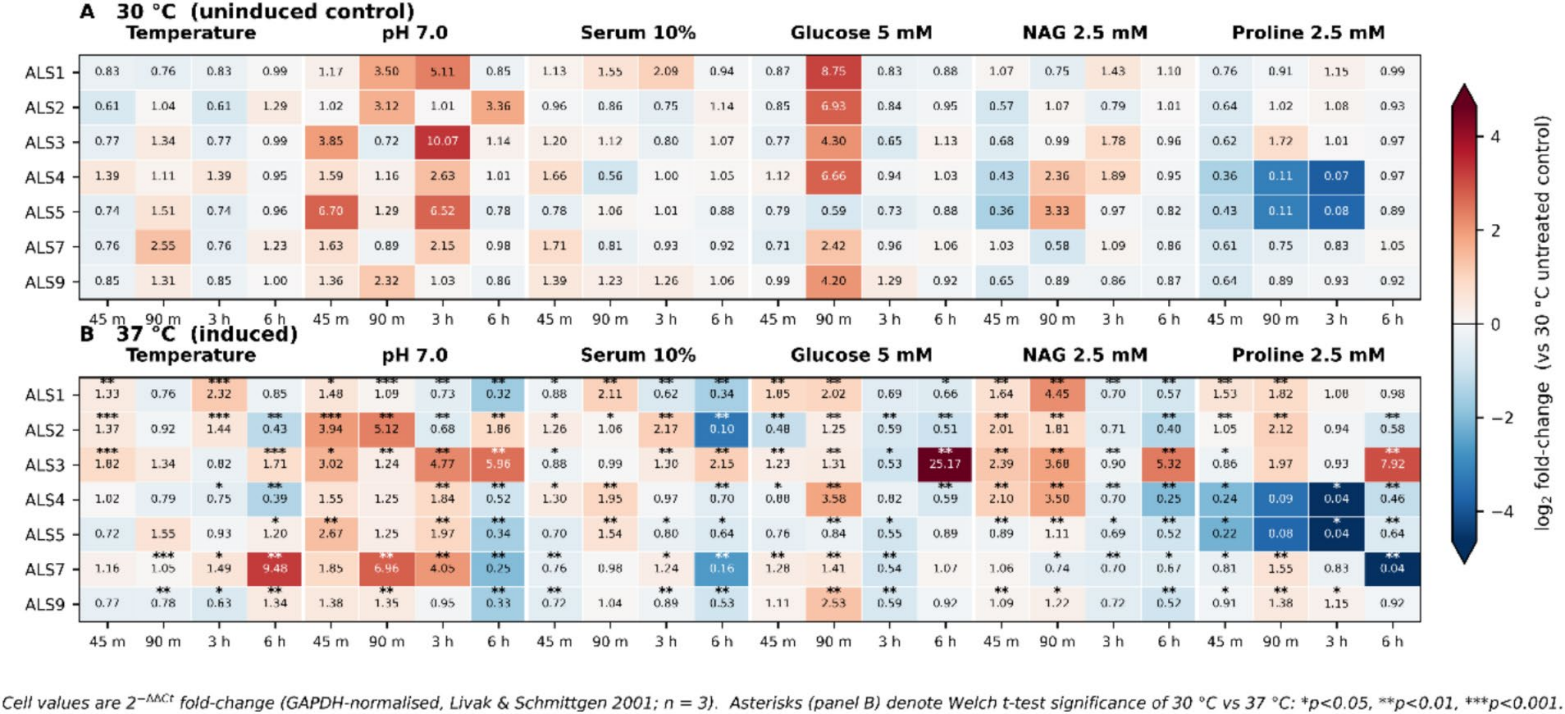
Heatmap of ALS gene expression in *C. albicans* across six morphogenic inducers and four post-induction time-points. Cell values are 2^-∆∆Ct^ fold-change (GAPDH-normalised, Livak and Schmittgen, 2001; *n* = 3). Asterisks (panel B) denote welch *t*-test significance of 30 °C vs. 37 °C: *p < 0.05, ** *p* < 0.01. *** *p* < 0.001.

**Figure 2:**
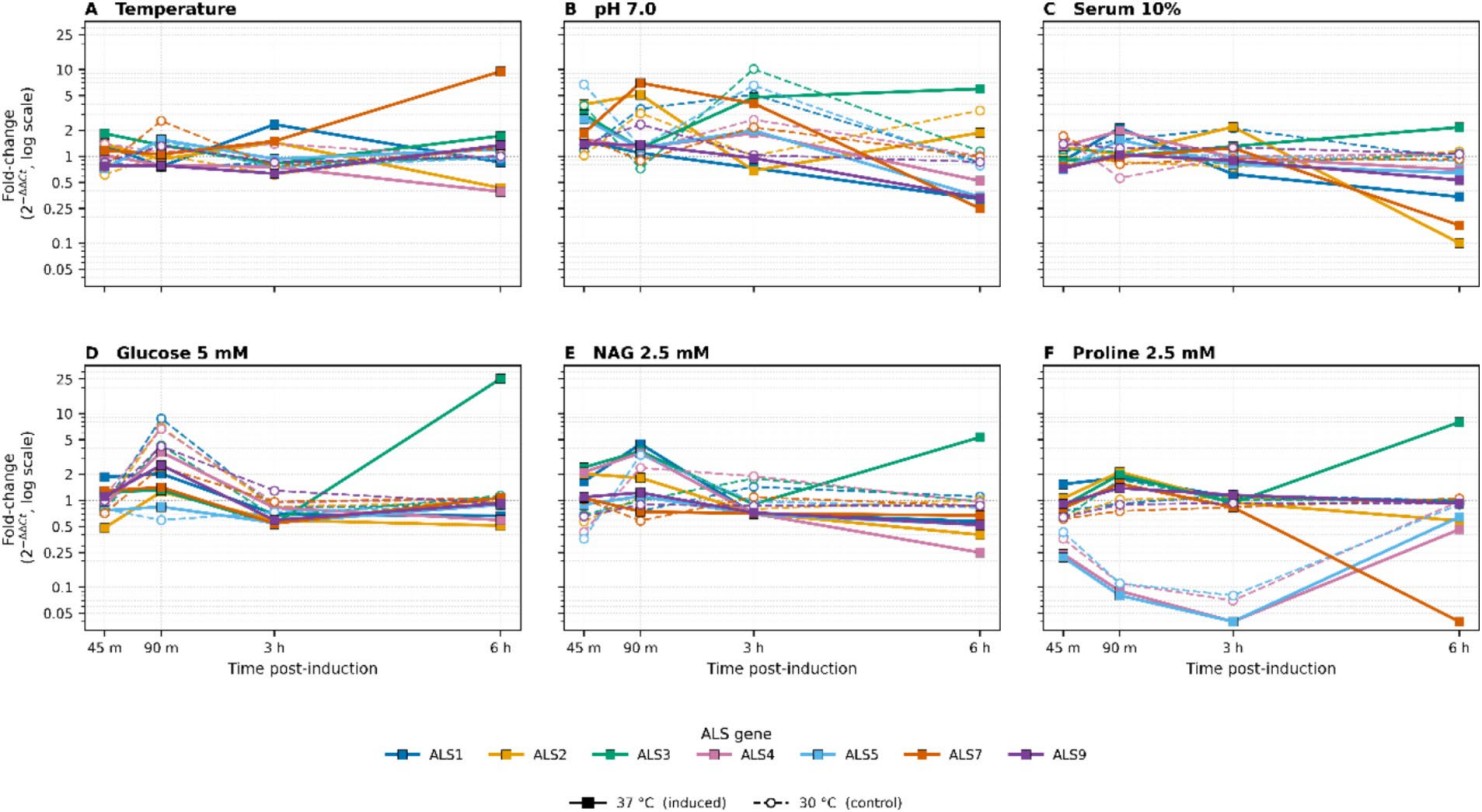
Time-course of ALS1-ALS9 expression in *C. albicans* under.

Exposure to 37 °C induced a temporally stratified pattern of *ALS* gene regulation (Table 3, Figures 1, 2). *ALS1* exhibited biphasic induction, peaking at 180 min (2.32 ± 0.12-fold; *p* = 0.003), consistent with its established role in sustained epithelial adhesion. *ALS2* was moderately induced early (1.37 ± 0.09-fold at 45 min; 1.44 ± 0.10-fold at 180 min), followed by pronounced repression at 360 min (0.43 ± 0.03; *p =* 0.001), indicating yeast-to-hypha transition-associated silencing. *ALS3* was induced at both early (1.82-fold at 45 min) and late time points (1.71-fold at 360 min), confirming its role as a sustained hyphal adhesin. The most striking observation was the dramatic late-stage induction of *ALS7* (9.48 ± 0.66-fold at 360 min; *p* < 0.001), suggesting a potential role in biofilm maturation or late-stage virulence processes. *ALS4* was significantly repressed at 360 min (0.39-fold), while *ALS5* and *ALS9* showed no significant thermal responsiveness.

**Table 3:** Expression pattern of *ALS* genes in response to temperature.

Gene	Condition	45 min	90 min	3 h	6 h
***ALS1***	**30 °C**	0.83 ± 0.06**	0.76 ± 0.05	0.83 ± 0.08***	0.99 ± 0.07
	**37 °C**	1.33 ± 0.11**	0.76 ± 0.05	2.32 ± 0.12***	0.85 ± 0.08
***ALS2***	**30 °C**	0.61 ± 0.09***	1.04 ± 0.12	0.61 ± 0.09***	1.29 ± 0.14**
	**37 °C**	1.37 ± 0.09***	0.92 ± 0.08	1.44 ± 0.10***	0.43 ± 0.03**
***ALS3***	**30 °C**	0.77 ± 0.11***	1.34 ± 0.09	0.77 ± 0.11	0.99 ± 0.06***
	**37 °C**	1.82 ± 0.11***	1.34 ± 0.09	0.82 ± 0.09	1.71 ± 0.09***
***ALS4***	**30 °C**	1.39 ± 0.26	1.11 ± 0.19	1.39 ± 0.26*	0.95 ± 0.08**
	**37 °C**	1.02 ± 0.08	0.79 ± 0.07	0.75 ± 0.05*	0.39 ± 0.04**
***ALS5***	**30 °C**	0.74 ± 0.12	**1.51 ± 0.22**	0.74 ± 0.12	0.96 ± 0.09*
	**37 °C**	0.72 ± 0.09	**1.55 ± 0.12**	0.93 ± 0.08	1.20 ± 0.10*
***ALS7***	**30 °C**	0.76 ± 0.28	2.55 ± 0.17***	0.76 ± 0.28*	1.23 ± 0.11**
	**37 °C**	1.16 ± 0.09	1.05 ± 0.10***	1.49 ± 0.09*	9.48 ± 0.66**
***ALS9***	**30 °C**	0.85 ± 0.08	1.31 ± 0.10**	0.85 ± 0.08*	1.00 ± 0.06**
	**37 °C**	0.77 ± 0.07	0.78 ± 0.06**	0.63 ± 0.05*	1.34 ± 0.09**

Values are mean fold-change (2^−ΔΔCt^) ± SD (*n* = 3 biological replicates) relative to GAPDH-normalized 30 °C untreated control (Livak and Schmittgen, 2001). Bold values: ≥1.5-fold induction or ≤0.5-fold repression. Superscript symbols denote statistical significance of 30 °C vs. 37 °C comparison by Welch’s *t*-test (*n* = 3 per group): *** *p* < 0.001; ** *p* < 0.01; * *p* < 0.05. Inducer: temperature (37 °C vs. 30 °C control; YEPD basal medium).

### 3.3 Neutral pH drives broad *ALS* induction with temperature-dependent modulation

Neutral pH (7.0) elicited the broadest transcriptional activation among all inducers tested (Table 4, Figures 1, 2). At 30 °C, *ALS3* showed a pronounced peak at 180 min (10.07 ± 0.70-fold), the highest yeast-phase induction recorded for any *ALS* gene in this study. *ALS5* was strongly activated early (6.70-fold at 45 min; 6.52-fold at 180 min), marking it as a pH-responsive, yeast-phase-preferential adhesin. At 37 °C, *ALS7* exhibited robust mid-phase induction (6.96-fold at 90 min; 4.05-fold at 180 min), followed by sharp repression at 360 min (0.25-fold). *ALS3* remained elevated throughout, culminating at 5.96-fold at 360 min. *ALS2* showed early induction (3.94–5.12-fold at 45–90 min) at 37 °C. These data indicate that pH acts as a potent modulator of *ALS* transcription, with distinct gene cohorts responding at different temperatures-a pattern consistent with activation via the Rim101 pH-sensing pathway.

**Table 4:** Expression pattern of *ALS* genes in response to pH.

Gene	Condition	45 min	90 min	3 h	6 h
***ALS1***	**30 °C**	1.17 ± 0.09*	3.50 ± 0.22***	5.11 ± 0.38**	0.85 ± 0.07**
	**37 °C**	1.48 ± 0.12*	1.09 ± 0.09***	0.73 ± 0.06**	0.32 ± 0.04**
***ALS2***	**30 °C**	1.02 ± 0.14***	3.12 ± 0.20**	1.01 ± 0.08**	3.36 ± 0.24**
	**37 °C**	3.94 ± 0.28***	5.12 ± 0.35**	0.68 ± 0.06**	1.86 ± 0.15**
***ALS3***	**30 °C**	3.85 ± 0.27*	0.72 ± 0.06**	10.07 ± 0.70**	1.14 ± 0.10**
	**37 °C**	3.02 ± 0.22*	1.24 ± 0.10**	4.77 ± 0.33**	5.96 ± 0.42**
***ALS4***	**30 °C**	**1.59 ± 0.12**	1.16 ± 0.09	2.63 ± 0.19**	1.01 ± 0.08**
	**37 °C**	**1.55 ± 0.13**	1.25 ± 0.11	1.84 ± 0.15**	0.52 ± 0.05**
***ALS5***	**30 °C**	6.70 ± 0.46**	1.29 ± 0.11	6.52 ± 0.45**	0.78 ± 0.07**
	**37 °C**	2.67 ± 0.19**	1.25 ± 0.11	1.97 ± 0.16**	0.34 ± 0.03**
***ALS7***	**30 °C**	**1.63 ± 0.11**	0.89 ± 0.08**	2.15 ± 0.16**	0.98 ± 0.09**
	**37 °C**	**1.85 ± 0.14**	6.96 ± 0.48**	4.05 ± 0.29**	0.25 ± 0.03**
***ALS9***	**30 °C**	1.36 ± 0.12	2.32 ± 0.17**	1.03 ± 0.09	0.86 ± 0.08**
	**37 °C**	1.38 ± 0.11	1.35 ± 0.12**	0.95 ± 0.08	0.33 ± 0.04**

Values are mean fold-change (2^−ΔΔCt^) ± SD (*n* = 3 biological replicates) relative to GAPDH-normalized 30 °C untreated control (Livak and Schmittgen, 2001). Bold values: ≥1.5-fold induction or ≤0.5-fold repression. Superscript symbols denote statistical significance of 30 °C vs. 37 °C comparison by Welch’s *t*-test (*n* = 3 per group): *** *p* < 0.001; ** *p* < 0.01; * *p* < 0.05. Inducer: neutral pH 7.0 (vs. 30 °C uninduced control).

### 3.4 Serum induces selective, temporally nuanced *ALS* regulation

Serum (10%) triggered the most selective *ALS* gene response among all inducers (Table 5, Figures 1, 2). At 37 °C, *ALS3* displayed delayed but sustained activation, reaching 2.15-fold by 360 min (*p* = 0.001) the most moderate *ALS3* induction among all 37 °C conditions. *ALS1* exhibited a biphasic pattern (2.11-fold at 90 min; 0.34-fold at 360 min), and *ALS2* peaked at 180 min (2.17-fold) before sharp repression (0.10-fold at 360 min). Strikingly, *ALS7* was profoundly repressed throughout (0.16-fold at 360 min; *p* < 0.001) in contrast to its strong induction under temperature and pH. *ALS9* was also progressively suppressed. These findings suggest that serum triggers a focused adhesin programme centred on *ALS3*-mediated invasion, while actively repressing other *ALS* family members, potentially to minimise immune detection during bloodstream infection.

**Table 5:** Expression pattern of *ALS* genes in response to serum.

Gene	Condition	45 min	90 min	3 h	6 h
***ALS1***	**30 °C**	1.13 ± 0.09*	1.55 ± 0.11**	2.09 ± 0.15**	0.94 ± 0.08**
	**37 °C**	0.88 ± 0.07*	2.11 ± 0.15**	0.62 ± 0.05**	0.34 ± 0.03**
***ALS2***	**30 °C**	0.96 ± 0.08*	0.86 ± 0.07*	0.75 ± 0.06**	1.14 ± 0.09**
	**37 °C**	1.26 ± 0.09*	1.06 ± 0.09*	2.17 ± 0.16**	0.10 ± 0.02**
***ALS3***	**30 °C**	1.20 ± 0.10*	1.12 ± 0.09	0.80 ± 0.07**	1.07 ± 0.09**
	**37 °C**	0.88 ± 0.07*	0.99 ± 0.08	1.30 ± 0.11**	2.15 ± 0.16**
***ALS4***	**30 °C**	1.66 ± 0.11*	0.56 ± 0.05**	1.00 ± 0.04	1.05 ± 0.08**
	**37 °C**	1.30 ± 0.10*	1.95 ± 0.14**	0.97 ± 0.08	0.70 ± 0.06**
***ALS5***	**30 °C**	0.78 ± 0.07	1.06 ± 0.09**	1.01 ± 0.08*	0.88 ± 0.07*
	**37 °C**	0.70 ± 0.06	1.54 ± 0.11**	0.80 ± 0.07*	0.64 ± 0.05*
***ALS7***	**30 °C**	1.71 ± 0.13**	0.81 ± 0.08	0.93 ± 0.08*	0.92 ± 0.08**
	**37 °C**	0.76 ± 0.06**	0.98 ± 0.08	1.24 ± 0.10*	0.16 ± 0.02**
***ALS9***	**30 °C**	1.39 ± 0.10**	1.23 ± 0.10	1.26 ± 0.10**	1.06 ± 0.08**
	**37 °C**	0.72 ± 0.06**	1.04 ± 0.09	0.89 ± 0.07**	0.53 ± 0.04**

Values are mean fold-change (2^−ΔΔCt^) ± SD (*n* = 3 biological replicates) relative to GAPDH-normalized 30 °C untreated control (Livak and Schmittgen, 2001). Bold values: ≥1.5-fold induction or ≤0.5-fold repression. Superscript symbols denote statistical significance of 30 °C vs. 37 °C comparison by Welch’s *t*-test (*n* = 3 per group): *** *p* < 0.001; ** *p* < 0.01; * *p* < 0.05. Inducer: 10% foetal bovine serum (vs. 30 °C uninduced control).

### 3.5 Glucose elicits the strongest *ALS3* induction among all conditions

Glucose (5 mM) induced the most dramatic single-gene response observed in this study (Table 6, Figures 1, 2). At 30 °C, a transient burst of expression was observed at 90 min for multiple *ALS* genes: *ALS1* (8.75-fold), *ALS2* (6.93-fold), *ALS4* (6.66-fold), *ALS3* (4.30-fold), and *ALS9* (4.20-fold). This coordinated burst was not sustained; by 180 min, expression had returned to near-baseline levels. At 37 °C, *ALS3* displayed extraordinary late-phase upregulation, reaching 25.17 ± 1.76-fold at 360 min (*p* < 0.001) the highest induction recorded for any ALS gene under any condition in this study. In contrast, *ALS2* was persistently repressed, and *ALS5* remained unresponsive throughout. These data highlight a tightly regulated, glucose-responsive transcriptional programme in which early yeast-phase colonisation adhesins give way to a dominant *ALS3*-driven invasive programme at host temperature.

**Table 6:** Expression pattern of *ALS* genes in response to glucose.

Gene	Condition	45 min	90 min	3 h	6 h
***ALS1***	**30 °C**	0.87 ± 0.07**	8.75 ± 0.61**	0.83 ± 0.07	0.88 ± 0.07*
	**37 °C**	1.85 ± 0.13**	2.02 ± 0.14**	0.69 ± 0.06	0.66 ± 0.05*
***ALS2***	**30 °C**	0.85 ± 0.07**	6.93 ± 0.48**	0.84 ± 0.07**	0.95 ± 0.08**
	**37 °C**	0.48 ± 0.05**	1.25 ± 0.10**	0.59 ± 0.05**	0.51 ± 0.04**
***ALS3***	**30 °C**	0.77 ± 0.06**	4.30 ± 0.30**	0.65 ± 0.05*	1.13 ± 0.09**
	**37 °C**	1.23 ± 0.10**	1.31 ± 0.11**	0.53 ± 0.04*	25.17 ± 1.76**
***ALS4***	**30 °C**	1.12 ± 0.09*	6.66 ± 0.47**	0.94 ± 0.08	1.03 ± 0.08**
	**37 °C**	0.88 ± 0.07*	3.58 ± 0.25**	0.82 ± 0.07	0.59 ± 0.05**
***ALS5***	**30 °C**	0.79 ± 0.07	0.59 ± 0.05**	0.73 ± 0.06*	0.88 ± 0.07
	**37 °C**	0.76 ± 0.06	0.84 ± 0.07**	0.55 ± 0.05*	0.89 ± 0.07
***ALS7***	**30 °C**	0.71 ± 0.06**	2.42 ± 0.17**	0.96 ± 0.08**	1.06 ± 0.08
	**37 °C**	1.28 ± 0.10**	1.41 ± 0.12**	0.54 ± 0.05**	1.07 ± 0.09
***ALS9***	**30 °C**	0.99 ± 0.08	4.20 ± 0.29**	1.29 ± 0.10**	0.92 ± 0.07
	**37 °C**	1.11 ± 0.09	2.53 ± 0.18**	0.59 ± 0.05**	0.92 ± 0.07

Values are mean fold-change (2^−ΔΔCt^) ± SD (*n* = 3 biological replicates) relative to GAPDH-normalized 30 °C untreated control (Livak and Schmittgen, 2001). Bold values: ≥1.5-fold induction or ≤0.5-fold repression. Superscript symbols denote statistical significance of 30 °C vs. 37 °C comparison by Welch’s *t*-test (*n* = 3 per group): *** *p* < 0.001; ** *p* < 0.01; * *p* < 0.05. Inducer: 5 mM glucose (vs. 30 °C uninduced control).

### 3.6 *N*-Acetylglucosamine induces a biphasic, temperature-dependent response

NAG (2.5 mM) induced a biphasic ALS transcriptional response that was strongly temperature-dependent (Table 7, Figures 1, 2). At 37 °C, *ALS1* peaked sharply at 90 min (4.45 ± 0.31-fold; *p* < 0.001), coincident with germ tube formation, before declining to 0.57-fold by 360 min. *ALS3* showed early activation (3.68-fold at 90 min) with a secondary spike at 360 min (5.32 ± 0.37-fold; *p* < 0.001), consistent with its dual roles in germ tube adhesion and subsequent hyphal invasion. *ALS4* was transiently induced (3.50-fold at 90 min) but strongly repressed by 360 min (0.25-fold). At 30 °C, most *ALS* genes remained at baseline, with the exception of *ALS5* (3.33-fold at 90 min) and *ALS4* (2.36-fold at 90 min).

**Table 7:** Expression pattern of *ALS* genes in response to NAG.

Gene	Condition	45 min	90 min	3 h	6 h
***ALS1***	**30 °C**	1.07 ± 0.09**	0.75 ± 0.07**	1.43 ± 0.11**	1.10 ± 0.09**
	**37 °C**	1.64 ± 0.13**	4.45 ± 0.31**	0.70 ± 0.06**	0.57 ± 0.05**
***ALS2***	**30 °C**	0.57 ± 0.05**	1.07 ± 0.09**	0.79 ± 0.07	1.01 ± 0.09**
	**37 °C**	2.01 ± 0.16**	1.81 ± 0.14**	0.71 ± 0.06	0.40 ± 0.04**
***ALS3***	**30 °C**	0.68 ± 0.06**	0.99 ± 0.08**	1.78 ± 0.13**	0.96 ± 0.08**
	**37 °C**	2.39 ± 0.18**	3.68 ± 0.26**	0.90 ± 0.08**	5.32 ± 0.37**
***ALS4***	**30 °C**	0.43 ± 0.04**	2.36 ± 0.18**	1.89 ± 0.14**	0.95 ± 0.08**
	**37 °C**	2.10 ± 0.17**	3.50 ± 0.25**	0.70 ± 0.06**	0.25 ± 0.03**
***ALS5***	**30 °C**	0.36 ± 0.03**	3.33 ± 0.24**	0.97 ± 0.08*	0.82 ± 0.07**
	**37 °C**	0.89 ± 0.08**	1.11 ± 0.10**	0.69 ± 0.06*	0.52 ± 0.04**
***ALS7***	**30 °C**	1.03 ± 0.09	0.58 ± 0.05*	1.09 ± 0.10**	0.86 ± 0.08*
	**37 °C**	1.06 ± 0.09	0.74 ± 0.07*	0.70 ± 0.06**	0.67 ± 0.06*
***ALS9***	**30 °C**	0.65 ± 0.06**	0.89 ± 0.08*	0.86 ± 0.08	0.87 ± 0.08**
	**37 °C**	1.09 ± 0.10**	1.22 ± 0.11*	0.72 ± 0.06	0.52 ± 0.05**

Values are mean fold-change (2^−ΔΔCt^) ± SD (*n* = 3 biological replicates) relative to GAPDH-normalized 30 °C untreated control (Livak and Schmittgen, 2001). Bold values: ≥1.5-fold induction or ≤0.5-fold repression. Superscript symbols denote statistical significance of 30 °C vs. 37 °C comparison by Welch’s *t*-test (*n* = 3 per group): *** *p* < 0.001; ** *p* < 0.01; * *p* < 0.05. Inducer: 2.5 mM N-acetylglucosamine, NAG (vs. 30 °C uninduced control).

### 3.7 Proline drives late-stage *ALS3* upregulation

Proline (2.5 mM) induced temperature-dependent *ALS* gene modulation with minimal effects at 30 °C (Table 8, Figures 1, 2). At 37 °C, several *ALS* genes were transiently activated during the first 90 min: *ALS1* (1.82-fold), *ALS2* (2.12-fold), *ALS4* (1.81-fold), and *ALS5* (1.81-fold). Most of these genes were repressed by 360 min. *ALS3* stood out with a dramatic late-stage surge (7.92 ± 0.55-fold at 360 min; *p* < 0.001). *ALS7* was profoundly repressed by 360 min (0.04-fold; *p* < 0.001), suggesting active silencing during proline-induced filamentation. These patterns indicate that proline activates an early adhesion programme followed by sustained *ALS3*-driven invasive growth.

**Table 8:** Expression pattern of *ALS* genes in response to proline.

Gene	Condition	45 min	90 min	3 h	6 h
***ALS1***	**30 °C**	0.76 ± 0.07**	0.91 ± 0.08**	1.15 ± 0.10	0.99 ± 0.09
	**37 °C**	1.53 ± 0.12**	1.82 ± 0.13**	1.08 ± 0.09	0.98 ± 0.09
***ALS2***	**30 °C**	0.64 ± 0.06**	1.02 ± 0.09**	1.08 ± 0.09	0.93 ± 0.08**
	**37 °C**	1.05 ± 0.09**	2.12 ± 0.15**	0.94 ± 0.08	0.58 ± 0.05**
***ALS3***	**30 °C**	0.62 ± 0.06*	**1.72 ± 0.14**	1.01 ± 0.09	0.97 ± 0.08**
	**37 °C**	0.86 ± 0.07*	**1.97 ± 0.14**	0.93 ± 0.08	7.92 ± 0.55**
***ALS4***	**30 °C**	0.36 ± 0.04	0.11 ± 0.01	0.07 ± 0.01	0.97 ± 0.09**
	**37 °C**	0.24 ± 0.03*	0.09 ± 0.01	0.04 ± 0.00*	0.46 ± 0.04**
***ALS5***	**30 °C**	0.43 ± 0.05	0.11 ± 0.01	0.08 ± 0.01	0.89 ± 0.09**
	**37 °C**	0.22 ± 0.03*	0.08 ± 0.01	0.04 ± 0.00*	0.64 ± 0.04**
***ALS7***	**30 °C**	0.61 ± 0.06*	0.75 ± 0.07**	0.83 ± 0.07	1.05 ± 0.09**
	**37 °C**	0.81 ± 0.07*	1.55 ± 0.11**	0.83 ± 0.07	0.04 ± 0.01**
***ALS9***	**30 °C**	0.64 ± 0.06*	0.89 ± 0.08**	0.93 ± 0.08*	0.92 ± 0.08
	**37 °C**	0.91 ± 0.08*	1.38 ± 0.10**	1.15 ± 0.10*	0.92 ± 0.08

Values are mean fold-change (2^−ΔΔCt^) ± SD (*n* = 3 biological replicates) relative to GAPDH-normalized 30 °C untreated control (Livak and Schmittgen, 2001). Bold values: ≥1.5-fold induction or ≤0.5-fold repression. Superscript symbols denote statistical significance of 30 °C vs. 37 °C comparison by Welch’s *t*-test (*n* = 3 per group): *** *p* < 0.001; ** *p* < 0.01; * *p* < 0.05. Inducer: 2.5 mM proline (vs. 30 °C uninduced control).

### 3.8 *In silico* analyses

#### 3.8.1 Promoter architecture

**Figure 3:**
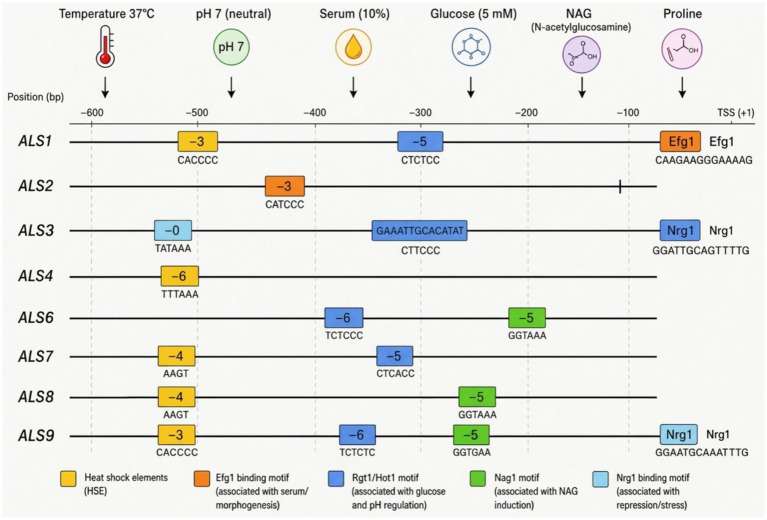
Promoter architecture model for the *ALS* gene family in *Candida albicans*, showing shared regulatory motifs and transcription factors (TFs) across *ALS* family.

*In silico* analysis of the 1 kb upstream regions revealed a modular regulatory design consistent with the observed expression stratification. Efg1 binding motifs were identified in the promoters of *ALS1*, *ALS3*, *ALS7*, and *ALS9*, aligning with their responsiveness to filament-inducing stimuli. Cph1 motifs were enriched in *ALS3* and *ALS4* promoters. The *ALS5* and *ALS9* promoters contained Rim101 motifs, consistent with their pH-responsive induction. The *ALS7* promoter was enriched for Tup1 motifs and lacked canonical Efg1/Cph1 sites, consistent with its tight repression under most conditions (Figures 3).

**Figure 4:**
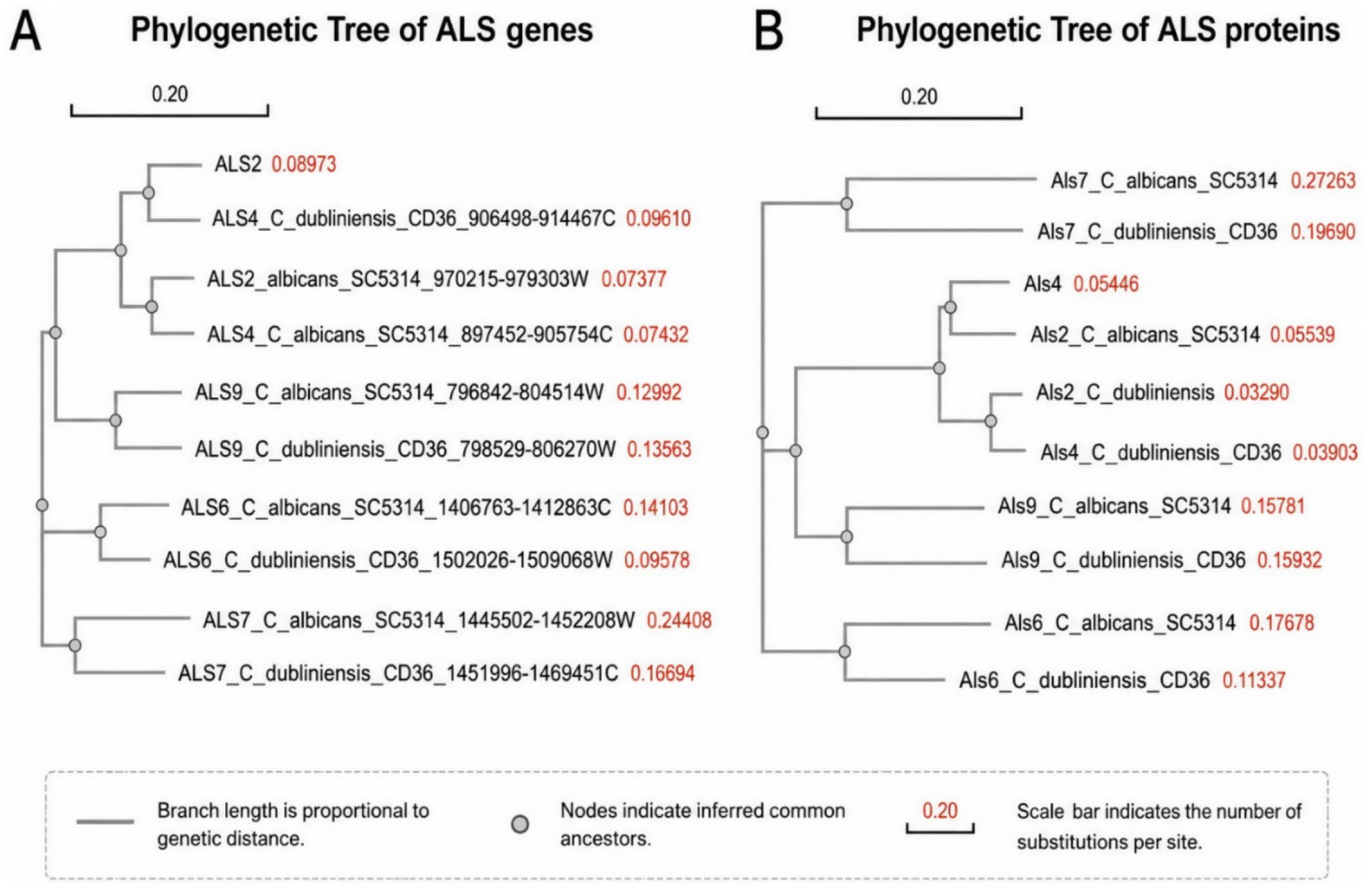
Phylogenetic analysis of *ALS* genes and proteins.

**Figure 5:**
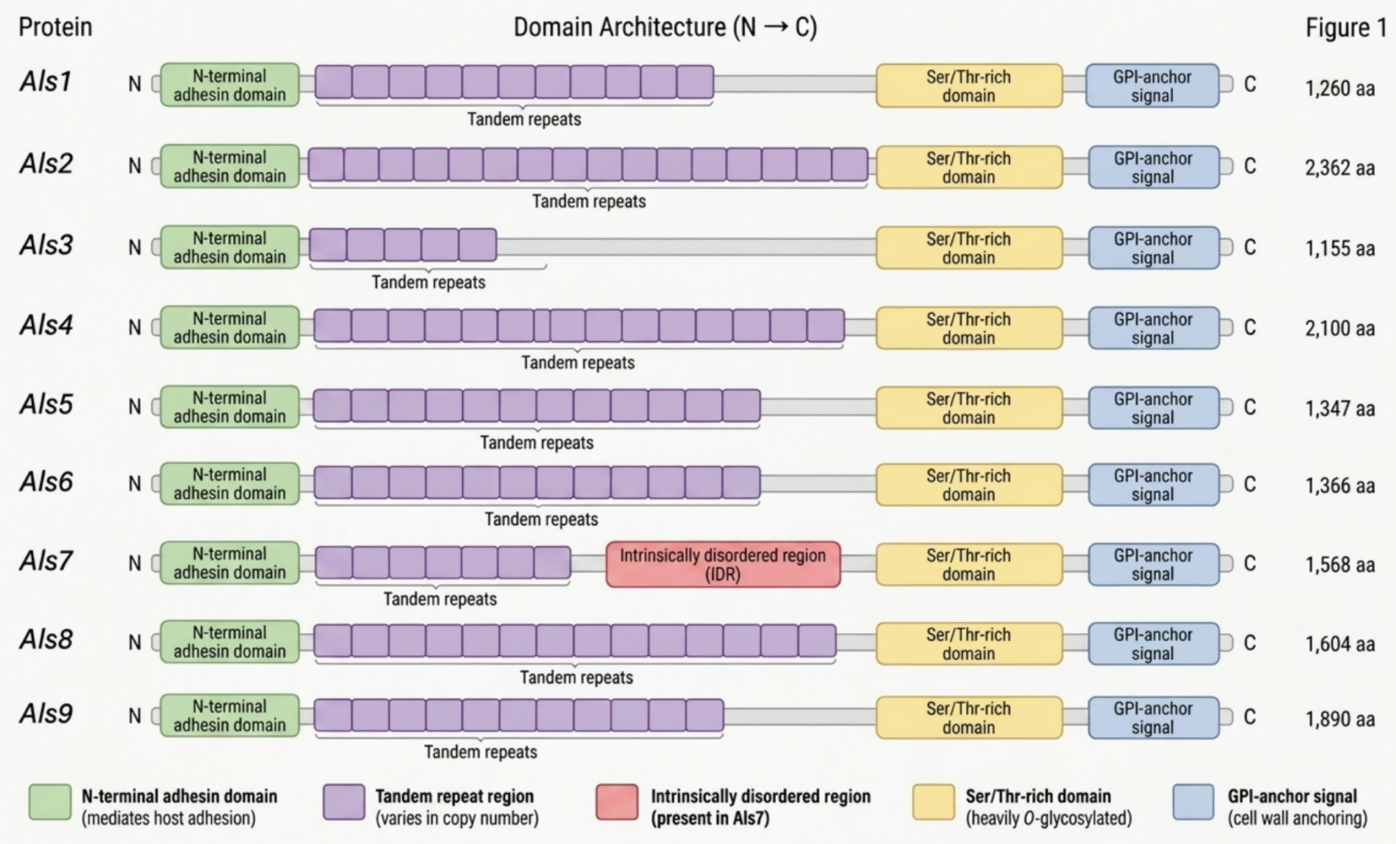
Domain architecture of *ALS* family. (All ALS proteins are GPI-anchored cell wall adhesins, Tandem repeat regions confer length variability and immune evasion, Als7p contains a unique intrinsically disordered region (IDR) between the repeat region and Ser/Thr-rich domain, which may confer structural flexibility and functional adaptation).

#### 3.8.2 Phylogenetic analysis

Neighbour-joining trees at both nucleotide and protein levels revealed close clustering of *ALS2* and *ALS4* across *C. albicans* and *C. dubliniensis*, indicating strong conservation. In contrast, *ALS7* was the most divergent (distance value 0.27263), followed by *ALS6* and *ALS9* (Figure 4).

#### 3.8.3 Protein interaction networks

STRING analysis confirmed that all ALS proteins form a tightly connected interaction cluster (enrichment *p* = 1.08 × 10^−16^), with shared involvement in biofilm formation (GO:0044391; FDR < 5 × 10^−17^), cell–cell adhesion (GO:0016337), and cell adhesion molecule binding (GO:0050839). A subset (*ALS1*–*ALS5*) exhibited fibronectin binding activity (GO:0001968).

#### 3.8.4 Domain architecture

All ALS proteins shared a conserved N-terminal adhesin domain (Pfam: PF11766). Tandem repeat regions varied in copy number and confidence: *ALS3* contained eight high-confidence repeats, while *ALS5* had low-confidence repeats. *ALS7* displayed an intrinsically disordered region (residues 801–1,030), and *ALS3* additionally carried bacterial-like adhesin domains (SUPERFAMILY SSF49401), potentially explaining its multifunctional adhesive and invasive properties (Figure 5).

## 4 Discussion

**Figure 6:**
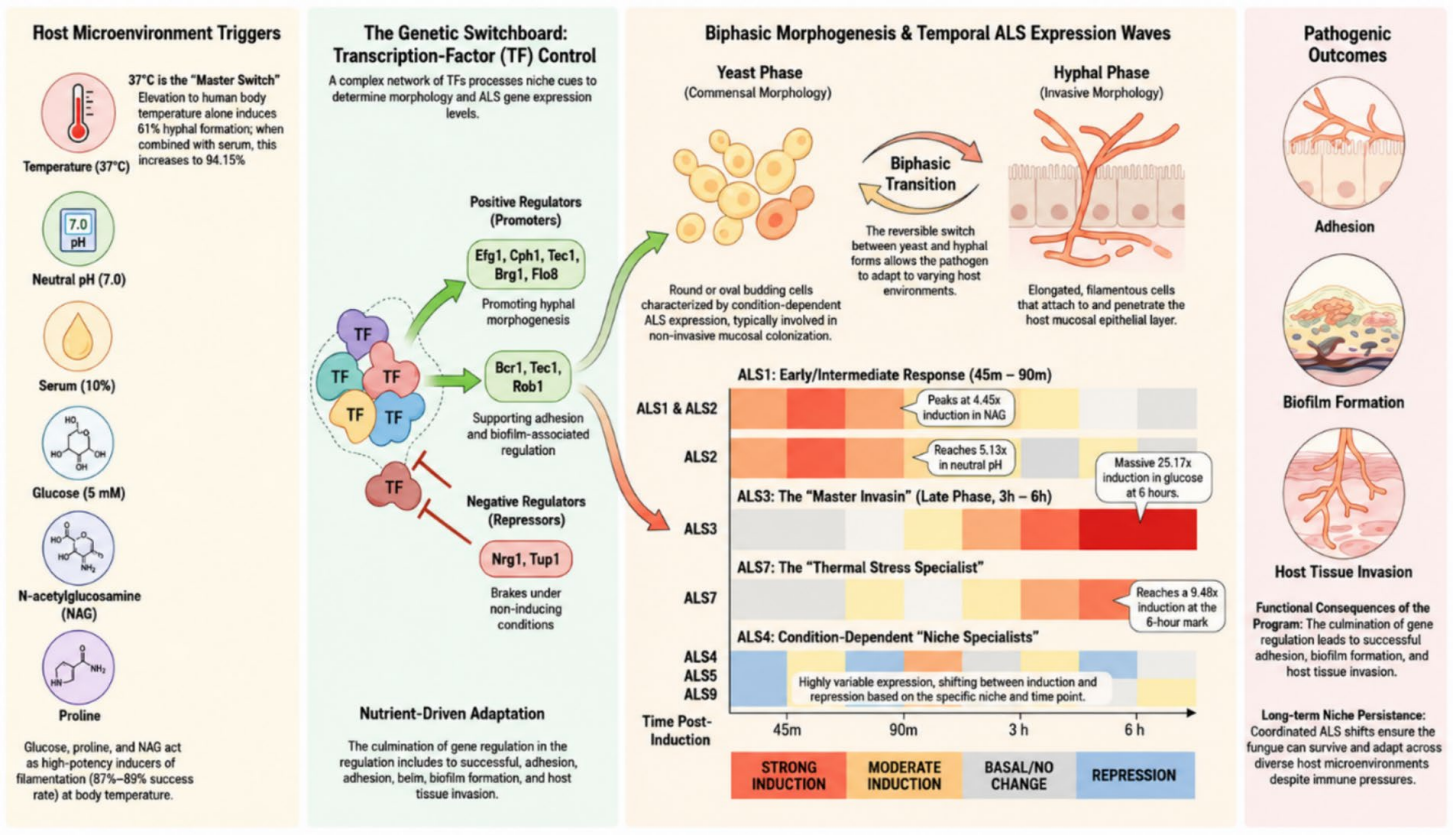
Niche-specific transcriptional regulation of biphasic *Candida albicans* morphology and *ALS* adhesin expression.

This study provides, to our knowledge, the first systematic temporal profiling of *ALS gene* family expression across six host-relevant environmental cues in *C. albicans*. Our findings reveal that the *ALS* family does not operate as a monolithic adhesion module; rather, individual members are deployed in a temporally stratified, condition-specific manner that reflects the diverse microenvironments encountered during infection (Figure 6).

### 4.1 A biphasic regulatory Programme governs ALS expression

**Figure 7:**
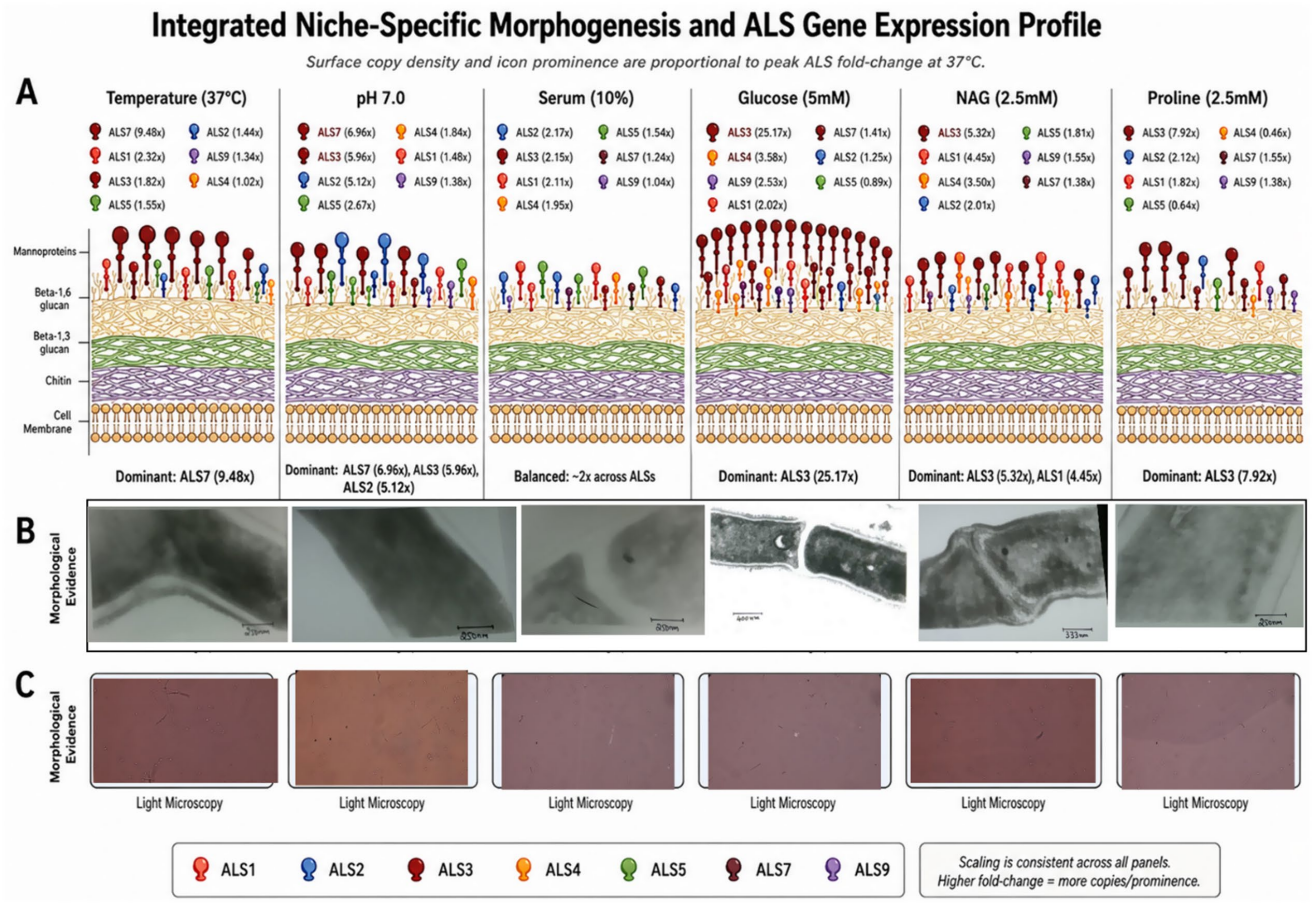
Morphological and ultrastructural responses of *Candida albicans* (ATCC 10231) to host-relevant environmental cues. **(A)** Conceptual model illustrating inducer-dependent cell wall remodeling and associated *ALS* gene expression patterns. **(B)** Representative transmission electron microscopy (TEM) images showing ultrastructural changes under the indicated inducing conditions. **(C)** Representative light microscopy images (40×) demonstrating inducer-specific morphological transitions. All conditions were evaluated at 37 °C and included temperature induction (37 °C), neutral pH, serum, glucose, *N*-acetylglucosamine (GlcNAc), and proline.

A central finding of this work is the identification of a biphasic temporal programme governing *ALS* expression during the yeast-to-hypha transition. Early-phase adhesins (*ALS1*, *ALS2*, and *ALS4*) were transiently induced within the first 45–90 min across multiple conditions, coinciding with initial germ tube emergence and early epithelial contact. These genes were subsequently silenced as hyphal elongation progressed, a pattern consistent with their established roles in yeast-phase adhesion and initial colonization (Fu et al., 2002; Zhao et al., 2005). The early burst of *ALS1* expression under glucose (8.75-fold at 90 min, 30 °C) and NAG (4.45-fold at 90 min, 37 °C) aligns with the known specificity of *Als1*p for fucose-linked *N*-acetylglucosamine residues on host cells (Donohue et al., 2011) (Figure 7).

Late-phase effectors, principally *ALS3* and *ALS7*, displayed sustained or escalating expression during mature hyphal development (3–6 h). The 25-fold induction of *ALS3* under glucose at 37 °C represents, to our knowledge, the highest *ALS gene* induction reported under defined conditions and is consistent with the known dependence of *ALS3* expression on the Efg1-dependent pathway and its regulation by the Nrg1/Tup1 repression complex (Argimón et al., 2007; Kadosh, 2019). The massive *ALS7* induction at 37 °C (9.48-fold) is noteworthy because *ALS7* has been considered a “quiet” member of the family (Hoyer and Hecht, 2000; Zhang et al., 2003). Our data suggest that its expression is not constitutively low but rather tightly restricted to specific thermal conditions, potentially related to its proposed contribution to amyloid-enriched biofilm matrices.

### 4.2 Inducer-specific patterns reveal niche-adapted Adhesin deployment

A striking outcome of this multi-inducer design is the demonstration that different environmental cues produce qualitatively distinct *ALS* expression signatures. Serum, the classical hyphal inducer, triggered the most selective response-activating *ALS3* while actively repressing *ALS7* and *ALS9*. In contrast, neutral pH elicited the broadest activation, inducing multiple *ALS gene*s simultaneously. Glucose and proline shared a common late-phase *ALS3* surge but differed in early-phase gene activation. These patterns suggest that *C. albicans* tailors its adhesin repertoire to specific host niches the focused *ALS3*-centric programme in serum may reflect adaptation to the bloodstream, where minimising surface antigen diversity could aid immune evasion, while the broad pH-responsive programme may serve mucosal colonisation, where multiple adhesion strategies are advantageous.

The preferential expression of *ALS5* at 30 °C under neutral pH (6.70-fold) versus its consistent repression at 37 °C establishes it as a yeast-phase, mucosal-niche adhesin. This is consistent with earlier reports of *ALS5* expression in yeast-phase cells in the reconstituted human epithelium model (Green et al., 2004) and supports the hypothesis that *ALS5* contributes to basal adhesion in body-temperature-independent mucosal niches.

### 4.3 Promoter architecture underpins expression stratification

Our *in silico* promoter analysis provides a mechanistic rationale for the observed expression patterns. The presence of tandem Efg1 and Nrg1 motifs in the *ALS1* and *ALS3* promoters is consistent with a dual-switch regulatory mechanism relief of Nrg1/Tup1 repression coupled with Efg1 activation during filamentation, as demonstrated by Zhao et al., 2004; Argimón et al., 2007. The enrichment of Rim101 motifs in *ALS5* and *ALS9* promoters correlates with the pH-responsive expression of these genes and places them downstream of the Rim101 pH-sensing pathway, which is known to regulate virulence gene expression at alkaline pH (Nobile et al., 2006; Glazier, 2022). The distinctive Tup1-enriched architecture of the *ALS7* promoter explains its tight basal repression and suggests that its dramatic induction under temperature stress may require chromatin remodelling events that override this repression.

### 4.4 Evolutionary divergence parallels functional specialisation

The phylogenetic conservation of *ALS2* and *ALS4* across *C. albicans* and *C. dubliniensis*, coupled with their similar expression profiles (both are early-phase, transiently induced adhesins), suggests functional redundancy and evolutionary maintenance of a core yeast-phase adhesion programme. In contrast, the marked divergence of *ALS7* at both nucleotide and protein levels correlates with its unique expression behaviour (dramatic thermal induction coupled with condition-specific repression) and its reported hypervariability at the tandem repeat locus (Zhang et al., 2003). This divergence may reflect adaptation to specialised niches or host-specific selective pressures.

### 4.5 Translational implications

Our findings have direct implications for anti-adhesion therapeutic strategies. *Als3p* emerged as the predominant virulence effector across virtually all conditions tested, with consistently strong late-phase induction. Given that *Als3*p functions as a multifunctional invasin mediating epithelial invasion, endothelial adhesion, and iron acquisition through ferritin binding (Phan et al., 2007; Liu and Filler, 2011), its broad and robust expression makes it the most attractive Candidate for monoclonal antibody-based interventions in disseminated candidiasis. Together, this study provides a meaningful transcriptional framework for understanding how *C. albicans* dynamically regulates *ALS* gene expression in response to host-relevant environmental cues. The observed transcriptional shifts are supported by our earlier findings showing cue-dependent changes in cell surface hydrophobicity (Shiradhone et al., 2018), as well as our unpublished data demonstrating altered adhesion and fluconazole susceptibility under temperature variation and NAG induction. Collectively, these findings strengthen the biological relevance of the model by linking morphogenesis, surface remodelling, antifungal response, and pathogenic adaptation. This work therefore provides a strong foundation for future validation using clinical isolates, ALS mutants, and *in vivo* infection models.

### 4.6 Study limitations

This study used the reference strain *Candida albicans* ATCC 10231, where our transcriptional findings align with earlier reports showing trigger-dependent changes in cell surface hydrophobicity and our unpublished adhesion data. Future validation using clinical isolates, *ALS* mutants, and *in vivo* virulence assays will further confirm the broader relevance and causal role of *ALS* regulation in pathogenicity.

## 5 Conclusion

This study presents the first comprehensive temporal mapping of *ALS gene* family regulation across six host-relevant environmental cues in *C. albicans*. We demonstrate that the *ALS* family is governed by a biphasic temporal programme in which early adhesins (*ALS1*, *ALS2*) mediate initial epithelial attachment, while late-phase effectors (*ALS3*, *ALS7*) drive tissue invasion and biofilm maturation. The inducer-specific nature of these programmes ranging from the focused, serum-driven *ALS3* response to the broad, pH-driven multi-gene activation reveals a level of regulatory sophistication that enables niche-specific virulence. Integration with *in silico* analyses of promoter architecture, evolutionary relationships, and protein interaction networks provides mechanistic depth to these observations. Collectively, these findings identify *ALS3* as a master virulence adhesin and could be a prime therapeutic target, while establishing *ALS5* and *ALS7* as promising *Candida*tes for niche-specific anti-adhesion interventions (Figure 6). However, these targets need to be validated using clinical isolates, *ALS* mutants and in murine infection models and explore inhibitors of the Efg1-*ALS3* regulatory axis as a foundation for next-generation antifungal strategies.

## Data Availability

The datasets presented in this study can be found in online repositories. The names of the repository/repositories and accession number(s) can be found in the article/supplementary material.
